# Rapid flow cytometric diagnosis of XIAP deficiency

**DOI:** 10.1111/pai.70284

**Published:** 2026-01-20

**Authors:** Ryosuke Wakatsuki, Madoka Nishimura, Dan Tomomasa, Shuhei Takahashi, Kyogo Suzuki, Koji Kawaguchi, Ryutaro Saura, Shota Inoue, Ichiro Takeuchi, Katsuhiro Arai, Masanaka Sugiyama, Yuta Narishige, Akira Oshima, Miyuki Tsumura, Satoshi Okada, Akihiro Hoshino, Masatoshi Takagi, Hirokazu Kanegane

**Affiliations:** ^1^ Department of Pediatrics and Developmental Biology, Graduate School of Medical and Dental Sciences Institute of Science Tokyo Tokyo Japan; ^2^ Department of Pediatrics, Graduate School of Medical Sciences Kumamoto University Kumamoto Japan; ^3^ Department of Hematology and Oncology Shizuoka Children's Hospital Shizuoka Japan; ^4^ Department of Gastroenterology, Nutrition, and Endocrinology Osaka Women's and Children's Hospital Osaka Japan; ^5^ Department of Hematology/Oncology Osaka Women's and Children's Hospital Osaka Japan; ^6^ Division of Gastroenterology, Center for Pediatric Inflammatory Bowel Disease National Center for Child Health and Development Tokyo Japan; ^7^ Department of Hematology and Oncology Tokyo Metropolitan Children's Medical Center Tokyo Japan; ^8^ Department of Gastroenterology Miyagi Children's Hospital Sendai Miyagi Japan; ^9^ Department of Infectious Disease & Immunology Kanagawa Children's Medical Center Yokohama Japan; ^10^ Department of Pediatrics Hiroshima University Hospital Hiroshima Japan; ^11^ Department of Pediatrics Hiroshima University Graduate School of Biomedical and Health Sciences Hiroshima Japan; ^12^ Department of Child Health and Development, Graduate School of Medical and Dental Sciences Institute of Science Tokyo Tokyo Japan

**Keywords:** CD62L, flow cytometry, MDP, NOD2, XIAP deficiency

## Abstract

**Introduction:**

X‐linked inhibitor of apoptosis protein (XIAP) deficiency is an inborn error of immunity caused by pathogenic variants of *XIAP*. It presents diverse symptoms, including recurrent hemophagocytic lymphohistiocytosis and inflammatory bowel disease. Previous reports established a functional analysis method that quantitatively evaluates intracellular tumor necrosis factor‐alpha (TNF‐α) production capacity following muramyl dipeptide (MDP) stimulation using flow cytometry (MDP‐flow TNF‐α) for assessing XIAP deficiency. However, this method required 2 days to obtain results, which is a limitation.

**Method:**

We established a method to measure the downregulation of L‐selectin (CD62L) on the cell surface after MDP stimulation of monocytes and neutrophils from patients with XIAP deficiency using flow cytometry (MDP‐flow CD62L) within 4 h. Moreover, we also evaluated MDP‐flow CD62L in patients with XIAP deficiency after allogeneic hematopoietic cell transplantation (HCT) to evaluate its usefulness in functional analysis.

**Results:**

Six patients with *XIAP* variants, two with interleukin‐1 receptor‐associated kinase 4 deficiency, and healthy controls were analyzed. The mean percent inhibition of CD62L expression (%inhibition) was evaluated in monocytes and neutrophils. The mean inhibition rates of CD62L expression in monocytes and neutrophils from patients with XIAP deficiency were 5.96% and 6.20%, respectively, significantly lower than those from healthy controls (monocytes, 85.4%; neutrophils, 85.4%). Furthermore, in three patients with XIAP deficiency after HCT, the MDP‐flow CD62L was evaluated post‐HCT, confirming improvement in accordance with donor chimerism.

**Conclusion:**

In XIAP deficiency, MDP‐flow CD62L enabled faster functional analysis than MDP‐flow TNF‐α. These analyses are also useful for post‐HCT functional assessment.

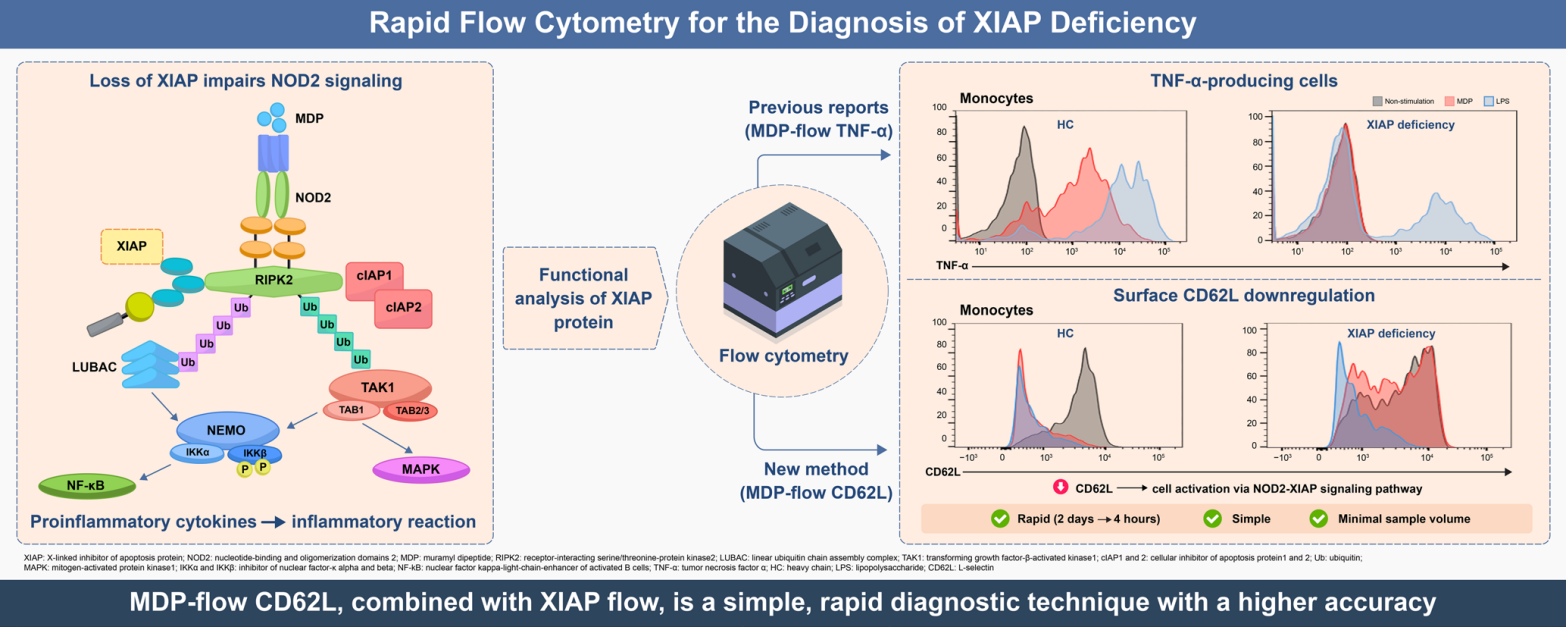

## Graphical abstract

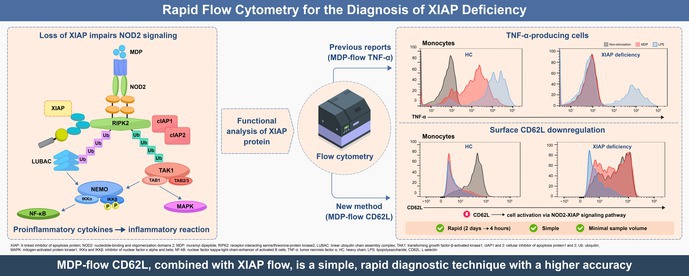

**Abbreviations**
CD62L: L‐selectin
HC: healthy control
HCT: hematopoietic cell transplantation
HLH: hemophagocytic lymphohistiocytosis
IBD: inflammatory bowel disease
IRAK4: IL‐1 receptor‐associated kinase 4
LPS: lipopolysaccharide
MDP: muramyl dipeptide
MFI: mean fluorescence intensity
TNF‐α: tumor necrosis factor‐α
XIAP: X‐linked inhibitor of apoptosis protein

**Key message**

Conventional functional analysis of XIAP deficiency requires complex assays over 2 days. We demonstrate that measuring CD62L downregulation after MDP stimulation by flow cytometry provides a simpler, faster assay requiring less sample and is also applicable for functional monitoring after hematopoietic cell transplantation in XIAP‐deficient patients.

## 1 INTRODUCTION

X‐linked inhibitor of apoptosis (XIAP) deficiency is a rare inborn error of immunity caused by the hemizygous variants of the *XIAP/BIRC*4 on the X chromosome.^1^ XIAP plays a crucial role in regulating cell survival and inflammation, and its loss of function leads to various symptoms. The most common symptom is hemophagocytic lymphohistiocytosis (HLH) observed in approximately 60% of patients.^2^ HLH in XIAP deficiency is often relatively mild but is characterized by frequent recurrence. Approximately 30% of patients present with inflammatory bowel disease (IBD). IBD in patients with XIAP deficiency is clinically and histologically similar to that in Crohn's disease.^2^ Genetic testing is necessary to diagnose XIAP deficiency. Recent reports indicate that deletions in exon 1, a non‐coding region, can cause XIAP deficiency,^3^ suggesting that sequence analysis of the coding region alone may not be sufficient for a definite diagnosis. Conventionally, screening for XIAP deficiency relies on measuring XIAP protein levels using flow cytometry. However, in some cases, the protein expression remains unchanged, particularly in patients with missense variants.^4^, ^5^, ^6^ Consequently, complementary functional analyses are necessary to diagnose XIAP deficiency. Previous research has reported a method for measuring XIAP deficiency function by adding muramyl dipeptide (MDP), which specifically stimulates the nucleotide‐binding and oligomerization domain 2 signaling pathway, to monocytes and then assessing their subsequent tumor necrosis factor‐alpha (TNF‐α) production capacity (MDP‐flow TNF‐α).^7^, ^8^ XIAP plays a crucial role in the nucleotide‐binding and oligomerization domain 2 signaling pathway, and XIAP deficiency causes a loss of responsiveness to MDP stimulation. This method was highly sensitive and specific, making it useful for diagnosing XIAP deficiency. However, it has drawbacks, including a two‐day assay and the depletion of monocytes during analytical procedures, which could obscure the results.^7^, ^8^ Recently, a flow cytometry method was reported to measure CD62L downregulation in monocytes and neutrophils after MDP stimulation by Rizzo, et al.^9^ We validated the method for performing MDP‐flow CD62L analysis in Japanese patients with XIAP deficiency. Additionally, we assessed whether function was recovered in patients with XIAP deficiency after allogeneic hematopoietic cell transplantation (HCT).

## 2 MATERIALS AND METHODS

### 2.1 Flow cytometry

#### 2.1.1 CD62L downregulation assay following MDP stimulation (MDP‐flow CD62L)

Blood samples were collected on the day of or before the experiment. Then, 100 μL of EDTA whole blood with either 1 μg/mL MDP, 0.25 μg/mL lipopolysaccharide (LPS), or no stimulus was incubated at 37°C for 2 h. Subsequently, the cells were stained with LIVE/DEAD Fixable Aqua (Thermo Fisher Scientific, Waltham, MA, USA), FITC‐conjugated CD62L (BD Biosciences, Franklin Lakes, NJ, USA), and AF700‐conjugated CD14 (BioLegend, San Diego, CA, USA). Hemolysis was performed using a hemolysis buffer (Dako, Glostrup, Denmark), and the cells were washed and analyzed using flow cytometry. Using FlowJo™ v10.6.2 Software (BD Life Sciences), mean fluorescence intensity (MFI) was measured, and % inhibition ((MFI_unstimulation − MFI_stimulation)/MFI_unstimulation × 100) was calculated.

#### 2.1.2 TNF‐α production assay following MDP stimulation (MDP‐flow TNF‐α)

Peripheral blood mononuclear cells were incubated overnight at 37°C and then divided into three conditions per sample: stimulation with 1 μg/mL MDP, stimulation with 0.25 μg/mL LPS, or no stimulation. GolgiPlug™ (BD Biosciences, Franklin Lakes, NJ, USA) was added to all conditions and incubated at 37°C for 2 h. After incubation, the cells were stained with LIVE/DEAD Fixable Aqua and FITC‐conjugated anti‐CD14 antibodies (BD Biosciences, Franklin Lakes, NJ, USA). The cells were then fixed and permeabilized using IntraPrep Permeabilization Reagent (Beckman Coulter, Brea, CA, USA). After washing, the cells were stained with APC‐conjugated anti‐TNF (BD Biosciences, Franklin Lakes, NJ, USA) and analyzed using flow cytometry. Using FlowJo™ v10.6.2 Software (BD Life Sciences), we measured the percentage of TNF‐α‐producing cells from the results.^7^, ^8^

#### 2.1.3 XIAP protein flow cytometry (XIAP flow)

Peripheral blood mononuclear cells were isolated, and 20 million cells were used for the assay. Cells were fixed with 1% paraformaldehyde for 30 min, then permeabilized with 0.5% saponin for 15 min. Subsequently, the cells were stained with a mouse anti‐XIAP antibody (Clone 48/hILP/XIAP; BD Biosciences, Franklin Lakes, NJ, USA). After washing, the cells were stained with an FITC‐conjugated goat anti‐mouse IgG1 antibody (Southern Biotech, Birmingham, AL, USA). XIAP protein expression was measured using flow cytometry, determining MFI in samples stained for XIAP and in isotype‐stained samples using FlowJo™ v10.6.2 Software.

### 2.2 Statistical analysis

Statistical analysis was performed using Python 3.13 64‐bit software (Python Software Foundation, USA). The Mann–Whitney *U* test was used to test for significant differences between the two groups. A *p*‐value <0.05 was considered statistically significant. For %inhibition in MDP‐flow CD62L, a cutoff value was set, and the sensitivity and specificity were calculated relative to it. The 95% confidence interval was estimated bilaterally using the Wilson score method (without continuity correction). The correlation between MDP‐flow TNF‐α and CD62L was evaluated using Pearson's correlation coefficient. A linear regression line with the coefficient of determination (*R* ^2^) is shown in the figure. Statistical significance was determined using a two‐tailed test.

## 3 RESULTS

### 3.1 Characteristics of patients with XIAP deficiency

We performed MDP flow CD62L in six Japanese patients with XIAP variants prior to HCT (Table 1) and compared them with a patient with IL‐1 receptor‐associated kinase 4 (IRAK4) deficiency and healthy controls (HC). Patients 2 and 5 correspond to patients 5 and 8, respectively, in a previous study.^8^ All patients were suspected of having XIAP deficiency due to recurrent HLH or IBD, and genetic variants were identified via target gene panel sequencing at the Kazusa DNA Research Institute in Japan. Although an *XIAP* variant (p.Glu350del) was identified in patient 6, in silico analyses suggested that it was nonpathogenic.

**TABLE 1:** Characteristics of patients with *XIAP* variants.

	*XIAP* variant	Age at onset	Age at diagnosis	Age at analysis	Family history	HLH^a^	IBD^b^	Hypogamma‐globulinemia	Spleno‐megaly	EBV^c^ infection	XIAP flow (MFI^d^)	MDP‐flow TNF‐α(TNF‐α producing cells (%))	MDP‐flow CD62L (Monocytes:%inhibition)
Patient1	c. 145C>T p.Arg49Ter	1m	9m	5y	−	+	−	−	−	−	445	0.52	9.43
Patient2	c.1420T>C p.Cys474Arg	9m	1y	4y	−	+	+	−	−	−	213	0.44	−11.3
Patient3	c.877G>C p.Gly293Arg	4y	12y	12y	−	+	+	+	−	−	764	1.77	11.7
Patient4	exon 3 deletion	3y	4y	4y	−	−	+	−	−	−	397	3.11	31.6
Patient5	c.1056+5G>C exon 4 skipping	1m	3m	3y	−	+	−	−	+	−	246	0.34	−11.7
Patient6	c.1048_1050delp.Glu350del	14d	−	10m	−	−	+	+	−	−	856	37.1	85.7

^a^ Hemophagocytic lymphohistiocytosis.

^b^ Inflammatory bowel disease.

^c^ Epstein–Barr virus.

^d^ Mean fluorescence intensity.

### 3.2 Downregulation of CD62L after MDP stimulation

**FIGURE 1:**
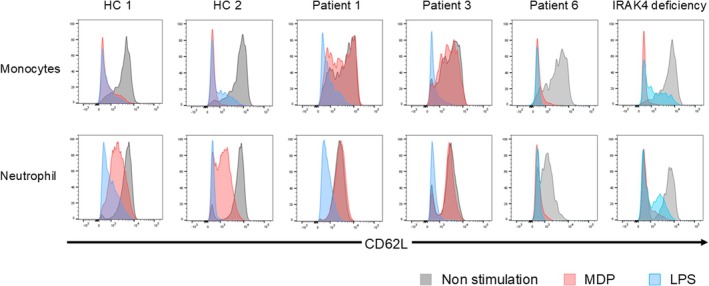
L‐selectin (CD62L) downregulation assay following muramyl dipeptide (MDP) stimulation (MDP‐flow CD62L). Flow cytometry showing downregulation of CD62L (MDP‐flow CD62L) in response to MDP or lipopolysaccharide (LPS) stimulation in two healthy controls (HC) and three patients with *XIAP* variants (patients 1, 3, and 6) and one patient with interleukin (IL)‐1 receptor‐associated kinase 4 (IRAK4) deficiency. Patient s1 and 4 showed no downregulation of CD62L even after MDP stimulation.

**FIGURE 2:**
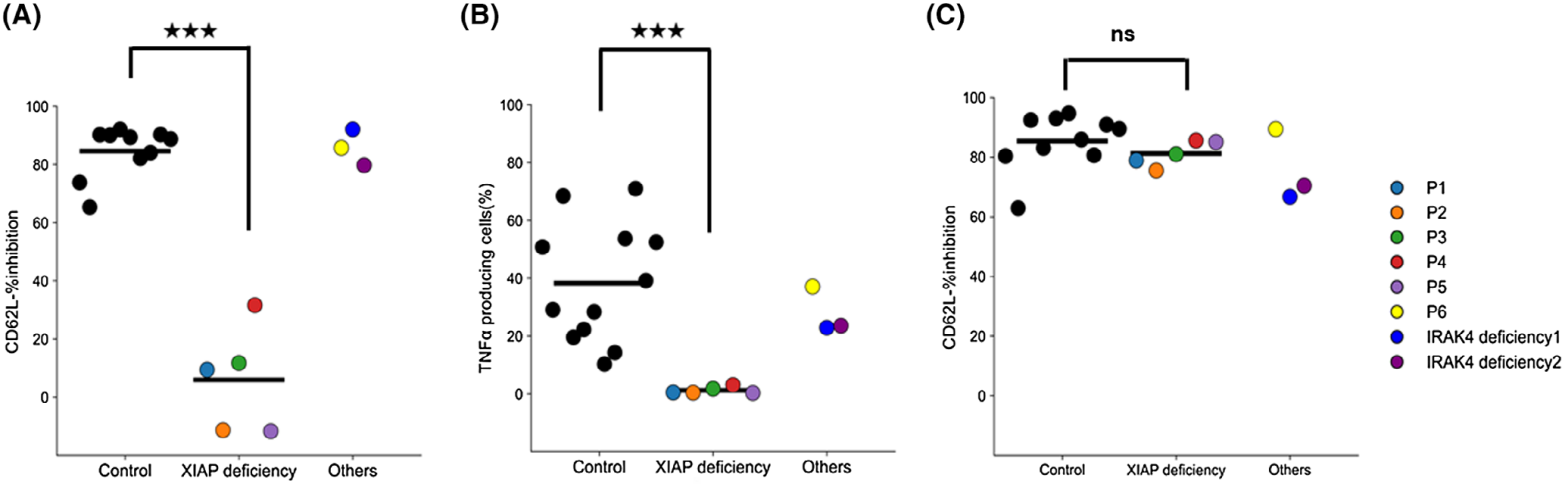
Results of muramyl dipeptide (MDP) flow. (A, C) Results of % inhibition in monocytes upon MDP (A) or lipopolysaccharide (LPS) (C) stimulation in MDP‐flow CD62L. MDP stimulation showed significantly lower % inhibition of X‐linked inhibitor of apoptosis protein (XIAP)‐deficient patients compared with that of HCs. (B) Results of tumor necrosis factor‐α (TNF‐α) producing cells (%) in monocytes upon MDP stimulation in MDP flow TNF‐α. MDP stimulation showed significantly higher % of producing cells in patients with XIAP deficiency than in HCs. ns indicates not significant; ^★★★^indicates *p* < .001.

Prior to analyzing patient samples, the assay conditions for MDP‐flow CD62L were evaluated using HC samples. Using the MDP‐flow TNF‐α conditions as a reference,^8^ we compared the concentrations of MDP and LPS and the incubation time (Figure S1). The MDP and LPS concentrations did not deviate significantly from the reference values. Regarding incubation time, since neutrophil % inhibition reached its maximum at 2 h, we set the incubation time to 2 h, consistent with the reference conditions. In patients with XIAP deficiency, no downregulation of CD62L was observed in monocytes or neutrophils following MDP stimulation. The % inhibition was significantly lower than that of HC, with a median of 9.43% and mean of 5.96% for monocytes (Figures 1 and 2A) and a mean of 6.99% and a mean of 6.20% for neutrophils (Figure S2A). Calculations indicated that setting the cutoff value at 35% would yield 100% sensitivity and specificity (sensitivity = 1.000, 95%CI [0.566, 1.000]; specificity = 1.000, 95%CI [0.722, 1.000]). Following LPS stimulation, % inhibition was high, even in XIAP‐deficient patients, showing no significant difference compared with HC.

Patient 6 showed high %inhibition after MDP stimulation (monocytes: 85.7%, neutrophils: 58.8%) (Figures 1 and 2). Similarly, in a patient with IRAK4 deficiency, %inhibition was lower than HC after LPS stimulation but comparable to HC after MDP stimulation (LPS stimulation: monocytes 66.7%, neutrophils: 59.4%) (Figures 1 and 2). We also examined whether the MDP‐flow CD62L results correlated with the MDP‐flow TNF‐α results reported in previous studies.^7^, ^8^ The correlation coefficient was 0.779, indicating a strong correlation between results (Figure S3).

### 3.3 Evaluation of XIAP protein function after HCT

**FIGURE 3:**
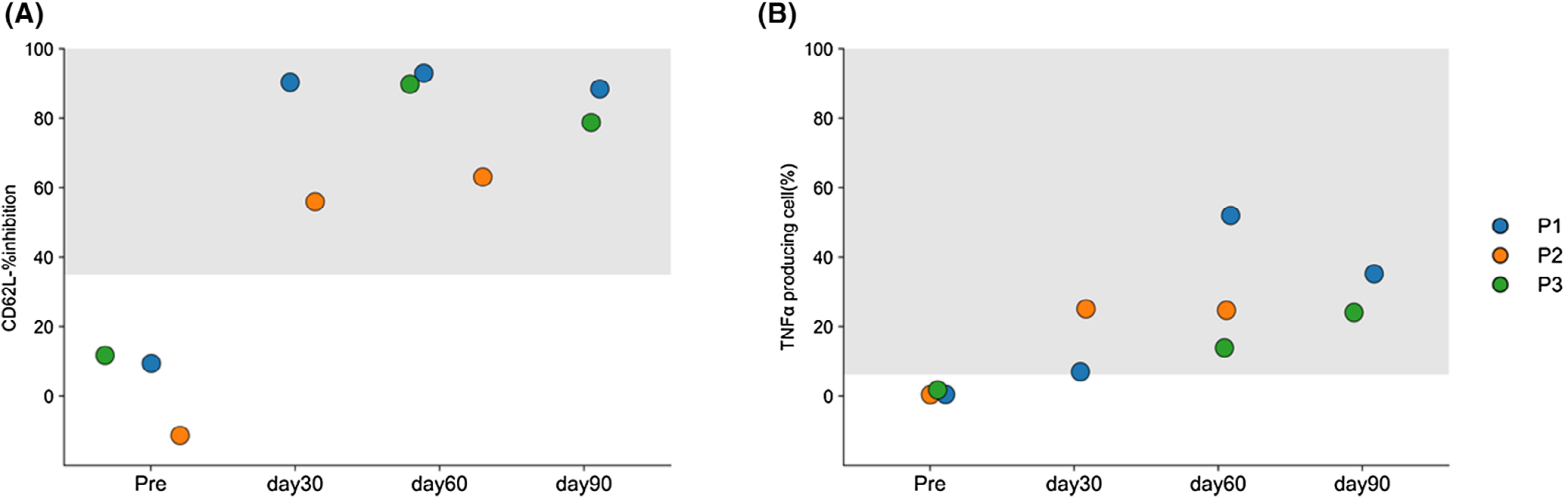
Results after hematopoietic cell transplantation in patients with XIAP deficiency. Results of MDP‐flow CD62L (A) and TNF‐α (B) in patient 1 before transplantation and at Days 30, 60, and 90 post‐transplantation, and in patient 2 before transplantation and at Days 30 and 60 post‐transplantation, and in patient 3 before transplantation and at days 60 and 90 post‐transplantation. The normal range is shown in gray. Improved response to MDP stimulation was observed after transplantation.

MDP flow CD62L and TNF‐α were performed before and after HCT in patients 1, 2, and 3. Measurements were taken on Days 30, 60, and 90 post‐transplantation for patient 1, and on Days 30 and 60 for patient 2, and days 60 and 90 for patient 3 (Figure 3, Table 2). These patients showed improvement in %inhibition of monocytes post‐transplantation compared with pre‐transplantation levels. Additionally, TNF‐α production capacity in monocytes post‐MDP stimulation and XIAP protein expression were similarly measured using flow cytometry. Improvements were observed in both patients. These results demonstrate that these three assays are useful for evaluating the function of post‐transplantation hematopoietic cells in patients with XIAP deficiency.

**TABLE 2:** Outcomes of hematopoietic cell transplantation in patients with XIAP deficiency.

Patient	Days	Chimerism (%)			XIAP flow (MFI)	MDP‐flow	
		Whole blood	Granulocyte	CD3^+^		TNF‐α(TNF‐α producing cells (%))	CD62L (Monocytes: %inhibition)
Patient 1	Pre‐transplantation				445	0.52	9.43
	Day 30	99.5	99.9	98.2	2139	7.03	90.4
	Day 60	98.1	99.8	96.6	931	52	93
	Day 90	99.2	98.3	97.1	757	35.2	88.5
Patient 2	Pre‐transplantation				213	0.44	−11.3
	Day 30	95.8	90.3	91.8	1386	25.1	56
	Day 60	99.2	96.1	90.9	454	24.7	63.1
Patient 3	Pre‐transplantation				764	1.77	11.7
	Day 60	99.8	98.9	100	1636	13.9	89.8
	Day 90	100	100	100	1486	24.1	78.8

*Note*: Chimerism in Patients 1, 2 and 3 before transplantation and at days 30, 60, and 90 post‐transplantation; MFI of XIAP protein using flow cytometry (XIAP Flow); % Inhibition of CD62L downregulation in monocytes stimulated with MDP.

## 4 DISCUSSION

This study reconfirmed that the MDP‐flow CD62L reported by A.D. Rizzo et al. enables simple and rapid functional evaluation of XIAP.^9^ CD62L evaluation required only antibody staining and hemolysis after MDP stimulation, completing the assay for a total of 4 h. This allows for a simpler and faster evaluation than MDP‐flow TNF‐α.^7^, ^8^ Furthermore, MDP‐flow CD62L enables testing with a minimal sample volume and demonstrates a strong correlation with MDP‐flow TNF‐α, making it a useful assay.

Previous reports have stated that measurement using fresh blood samples is essential because physiological downregulation of CD62L is observed in both monocytes and neutrophils.^9^ Although our study also observed physiological downregulation, we found that evaluation is feasible for monocytes and neutrophils using the “% inhibition” method—which compares CD62L levels to a baseline—if samples are collected and tested by the following day. This indicates that evaluation is possible not only at our facility but also at facilities within a distance allowing transport within 1 day.

Patient 6 had a p.Glu350del in the *XIAP* gene. However, according to ToMMo (JMorp database, Tohoku Medical Megabank Organization, Tohoku University), which indicates allele/genotype frequencies in Japanese populations, this variant was observed at a high frequency of 4.5%, suggesting it was likely a nonpathogenic variant. The results of MDP‐low CD62L also showed no difference from those of HC, confirming the exclusion of XIAP deficiency. Therefore, the evaluation of XIAP function enables diagnosis, including the determination of XIAP deficiency, and has proven useful even in cases where genetic testing alone is inconclusive.

IRAK4 plays a crucial role in the toll‐like receptor 4 signaling pathway, which is specific to LPS stimulation. In patients with IRAK4 deficiency, the response to LPS was reduced, whereas stimulation with MDP yielded results similar to those in HC. Measuring the LPS response in patients with IRAK4 deficiency also allows for evaluation using the same assay as that used in this study, enabling a rapid functional assessment similar to that for patients with XIAP deficiency.

Functional assessment of the XIAP protein, including MDP flow‐CD62L, was performed in patients with XIAP deficiency after HCT. Although the evaluation was conducted up to 3 months post‐transplantation, an improvement in XIAP protein function was observed, similar to the changes in chimerism. This demonstrated the usefulness of XIAP assessment as a post‐transplant evaluation tool. Following HCT, evaluating the disease status is often challenging because of various factors such as infections and graft‐versus‐host disease. Phenotypes similar to those observed in XIAP deficiency, such as post‐transplant‐related HLH, autoimmune diseases, and intestinal graft‐versus‐host disease, may occur. Functional assessment of the XIAP protein is particularly useful in such situations. Therefore, following HCT in patients with XIAP deficiency, it is necessary to evaluate not only donor chimerism but also XIAP protein function via MDP‐flow.

One limitation is the physiological downregulation of CD62L, as mentioned earlier. If a certain level of CD62L expression is not observed in the unstimulated state, it is difficult to evaluate its downregulation. This tendency was more pronounced in neutrophils, sometimes limiting the evaluability of specimens processed on the second day after blood collection. In contrast, monocytes were less affected, allowing for reliable evaluation with maintained reproducibility even after 2 days. Fortunately, in Japan, the specimen transport technology allows samples to reach our facility on the day after collection, thereby enabling evaluation across cases nationwide. Second, the number of patients with XIAP deficiency measured was relatively small (five), and the cutoff value presented here may be subject to change. XIAP deficiency is a rare disorder; therefore, defining precise cutoff values will likely require a large‐scale international multicenter case series. However, combining *XIAP* gene testing, XIAP flow, and MDP‐flow allows for a reliable diagnosis of XIAP deficiency in all cases, including those that might be missed by a single test. MDP‐flow CD62L is simple and can be used for screening alongside XIAP flow.

## 5 CONCLUSION

In this study, we used MDP‐flow CD62L reported by A.D. Rizzo et al. to evaluate the function of XIAP deficiency. This assay proved useful for functional analysis and was confirmed to be simple and rapid for both diagnosis and post‐HCT evaluation. When screening for XIAP deficiency, combining MDP‐flow CD62L with XIAP flow is expected to improve diagnostic accuracy.

## AUTHOR CONTRIBUTIONS

**Ryosuke Wakatsuki:** Data curation; formal analysis; writing – original draft. **Madoka Nishimura:** Formal analysis. **Dan Tomomasa:** Resources. **Shuhei Takahashi:** Resources. **Kyogo Suzuki:** Resources. **Koji Kawaguchi:** Resources. **Ryutaro Saura:** Resources. **Shota Inoue:** Resources. **Ichiro Takeuchi:** Resources. **Katsuhiro Arai:** Resources. **Masanaka Sugiyama:** Resources. **Yuta Narishige:** Resources. **Akira Oshima:** Resources. **Miyuki Tsumura:** Resources. **Satoshi Okada:** Supervision. **Akihiro Hoshino:** Supervision. **Masatoshi Takagi:** Supervision. **Hirokazu Kanegane:** Conceptualization; funding acquisition; writing – review and editing.

## FUNDING INFORMATION

This work was supported by the Japan Society for the Promotion of Science (JSPS) KAKENHI (grant number: 22K07887) awarded to H.K.

## CONFLICT OF INTEREST STATEMENT

The authors declare no conflicts of interest.

## ETHICS STATEMENT

Genetic analysis was performed after obtaining written informed consent from all patients. This study was performed in accordance with the Declaration of Helsinki and approved by the Ethics Committee of the Institute of Science Tokyo (G2019‐004).

## Supporting information


**Figure S1.** Reviewing conditions for MDP‐flow CD62L.
**Figure S2:** Results of MDP‐flow CD62L in neutrophils.
**Figure S3:** Correlation diagram of MDP‐flow CD62L and TNF‐α.
