# Supplementary material for: Rapid flow cytometric diagnosis of XIAP deficiency
Source: Pediatr Allergy Immunol. 2026 Jan 20;37(1):e70284. doi: 10.1111/pai.70284 (PMC12817240; doi:10.1111/pai.70284)
Supplement: Supplementary file 1 — Figure S1. Reviewing conditions for MDP‐flow CD62L. Figure S2: Results of MDP‐flow CD62L in neutrophils. Figure S3: Correlation diagram of MDP‐flow CD62L and TNF‐α. [file PAI-37-e70284-s001.docx]

Supplementary materials for "**Rapid flow cytometric diagnosis of XIAP deficiency**" in Pediatric Allergy and Immunology

Ryosuke Wakatsuki, Madoka Nishimura, Dan Tomomasa, Shuhei Takahashi, Kyogo Suzuki, Koji Kawaguchi, Ryutaro Saura, Shota Inoue, Ichiro Takeuchi, Katsuhiro Arai, Masanaka Sugiyama, Yuta Narishige, Akira Oshima, Miyuki Tsumura, Satoshi Okada, Akihiro Hoshino, Masatoshi Takagi, Hirokazu Kanegane

Corresponding author

Hirokazu Kanegane, M.D., Ph.D.

Department of Child Health and Development, Graduate School of Medical and Dental Sciences, Institute of Science Tokyo

1-5-45 Yushima, Bunkyo-ku, Tokyo 113-8519, Japan

E-mail: hkanegane.ped@tmd.ac.jp

**
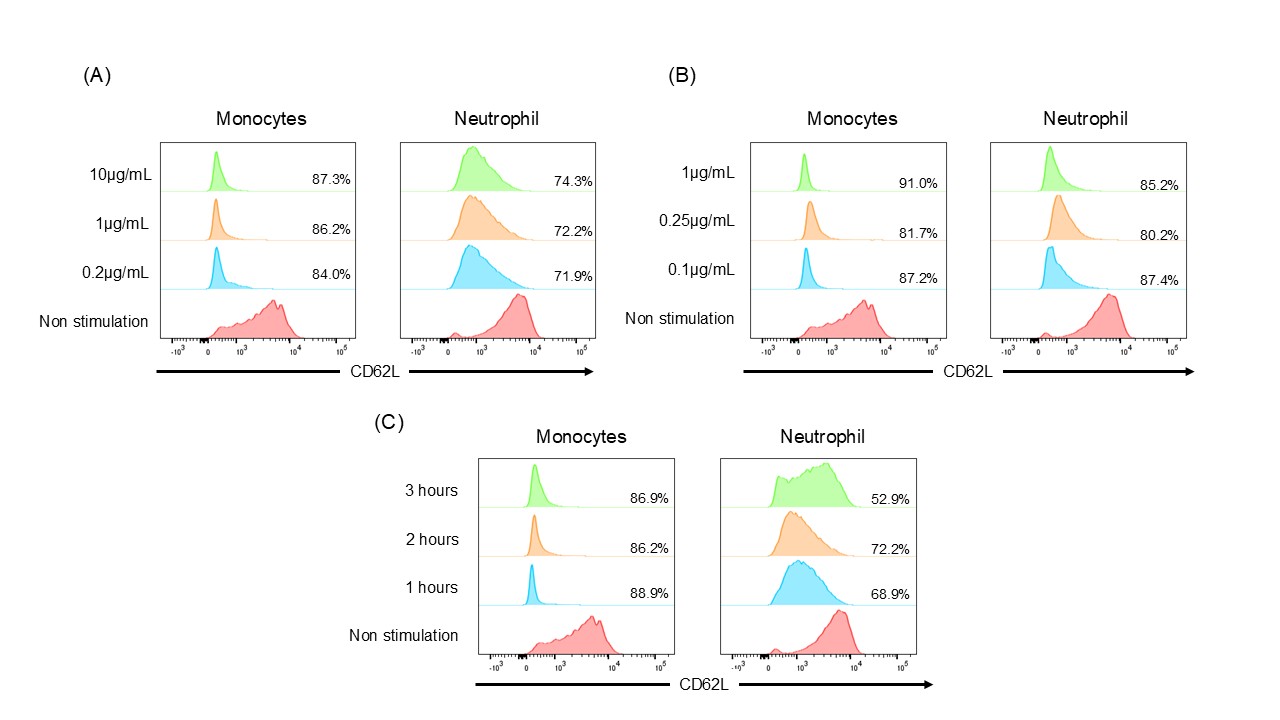
**

**Supplementary Figure 1. Reviewing conditions for MDP-flow CD62L**

For HC samples, we evaluated the conditions for the MDP-flow CD62L assay. Results from evaluating MDP (A) and LPS (B) concentrations (incubation time: 2 h) and from evaluating incubation time at MDP 1μg/mL (C). The MDP-flow TNF-α concentration and incubation time were used as reference points. The results at the reference concentration and time indicated no problms with the assays. The numbers in the box indicate the percentage inhibition.


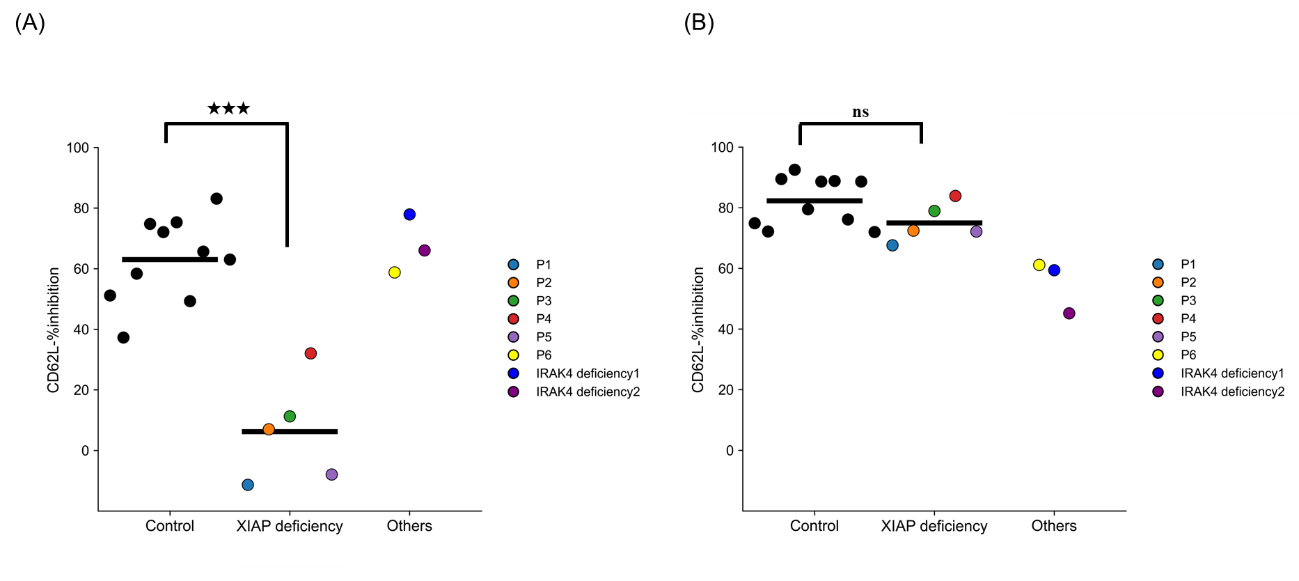


**Supplementary Figure 2: Results of MDP-flow CD62L in neutrophils**

Results of % inhibition in neutrophils upon MDP (A) or LPS (B) stimulation of MDP-flow CD62L. MDP stimulation resulted in significantly lower % inhibition in patients than in HC.


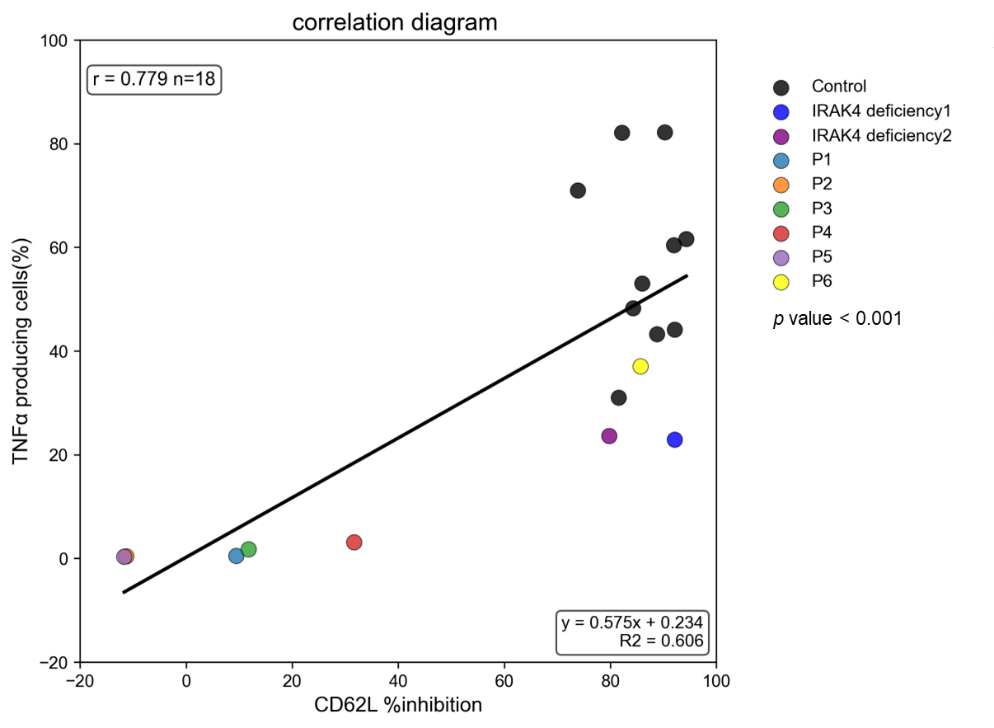


**Supplementary Figure 3: Correlation diagram of MDP-flow CD62L and TNF-α**

Correlation diagram of MDP-flow CD62L and TNF-α results. A strong correlation was observed between the results of two assays.
